# Visualization of Spatial Charge in Thermally Poled Glasses via Nanoparticles Formation

**DOI:** 10.3390/nano11112973

**Published:** 2021-11-05

**Authors:** Ekaterina Babich, Ekaterina Lubyankina, Vladimir Kaasik, Alexey Mozharov, Ivan Mukhin, Valentina Zhurikhina, Andrey Lipovskii

**Affiliations:** 1 Laboratory of Multifunctional Glassy Materials, World-Class Research Center “Advanced Digital Technologies”, Peter the Great St. Petersburg Polytechnic University, Polytechnicheskaya 29, 195251 St. Petersburg, Russia; babich.katherina@gmail.com (E.B.); vkaasik@yandex.ru (V.K.); lipovskii@mail.ru (A.L.); 2 Laboratory of Nanophotonics, Alferov University, Khlopina 8/3, 194021 St. Petersburg, Russia; 3 Scientific Educational Center “Physics and Technology of Heterogeneous Materials and Nanoheterostructures”, Institute of Physics and Mechanics, Peter the Great St. Petersburg Polytechnic University, Polytechnicheskaya 29, 195251 St. Petersburg, Russia; katylubyankina@gmail.com; 4 Laboratory of Optics of Heterogeneous Structures and Optical Materials, Alferov University, Khlopina 8/3, 194021 St. Petersburg, Russia; 5 Laboratory of Renewable Energy, Alferov University, Khlopina 8/3, 194021 St. Petersburg, Russia; alex000090@gmail.com (A.M.); imukhin@yandex.ru (I.M.); 6 Higher School of Engineering Physics, Institute of Electronics and Telecommunications, Peter the Great St. Petersburg Polytechnic University, Polytechnicheskaya 29, 195251 St. Petersburg, Russia

**Keywords:** metal nanoparticles, multicomponent glasses, thermal poling, spatial charge

## Abstract

It is shown for the first time that the vacuum poling of soda-lime silicate glass and the subsequent processing of the glass in a melt containing silver ions results in the formation of silver nanoparticles buried in the subanodic region of the glass at a depth of 800–1700 nm. We associate the formation of nanoparticles with the transfer of electrons from negatively charged non-bridging oxygen atoms to silver ions, their reduction as well as their clustering. The nanoparticles do not form in the ion-depleted area just beneath the glass surface, which indicates the absence of a spatial charge (negatively charged oxygen atoms) in this region of the vacuum-poled glass. In consequence, the neutralization of the glass via switching of non-bridging oxygen bonds to bridging ones, which leads to the release of oxygen, should occur in parallel with the shift of calcium, magnesium, and sodium ions into the depth of the glass.

## 1. Introduction

Recently, thermal poling of glasses, which involves applying a DC voltage to heated glass plates and cooling the glass under the applied voltage, has attracted new interest. This is mainly due to results obtained in studies of poled multicomponent glasses, which essentially differ from long-time studied poling of silica glasses. Contrary to a silica glass, multicomponent glasses demonstrate poling-induced altering of surface wetting [1] and reactivity [2,3] allowing for the formation of given 2D relief structures via etching [4,5], a change in refractive index [6,7], hardness [8,9], volume [10], and crystallization ability [11,12]. Additionally, extremely high second-order optical nonlinearity of a poled multicomponent glass has recently been registered [13]. In general, these phenomena are because of a strong structural and compositional modification of the subanodic region of multicomponent glasses by poling [14,15]. Concerning the nonlinearity, it is mainly associated with the spatial charge accumulated and frozen in the subanodic region of poled glasses [16], although the orientation of dipoles in glasses is also considered a source of nonlinearity [17,18]. Contrary to the orientational phenomena, the presence of spatial charge was experimentally confirmed using Lipp studies [15], the measurements of electric potential [19,20], and, in corona-poled glasses, by the observation of scattered electrons [21]. In the case of open anode poling configuration, which allows positively charged species from the surrounding to penetrate the glass, a difference in mobilities of alkali cations in the glass and penetrating cations (hydronium ions generated from water vapors if poling performed in air atmosphere [22]) is responsible for the formation of spatial charge. In the case of close anode poling configuration, which prevents the penetration of any species into the subanodic glass region, the spatial charge arises due to the depletion of the subanodic region of the glass with mobile monovalent and bivalent cations of the glass modifiers [15]. In the second case, a switching of non-bridging oxygen bonds to bridging ones with the release of oxygen also takes place [14,23], which results in partial compensation of the arising spatial charge. This compensation leads to the formation of a poling-modified glass layer, which is much thicker than what von Hippel formalism predicts [24]. Presently the knowledge about the behavior of the spatial charge in poled glasses is poor because of insufficient experimental data related. In this paper, we demonstrate for the first time that the introduction of silver ions in glasses poled in closed anode mode results in their reduction via receiving of negative charge and subsequent clustering to silver nanoparticles within the region buried in the glass and approximately coinciding with the region of significant concentration gradients of glass modifiers.

## 2. Materials and Methods

In the experiments, we used 1-mm-thick soda-lime glass slides (“Menzel”) purchased from Agar Scientific Ltd. (Stansted, UK) [25]. The glass composition is presented in Table 1.

We thermally poled the glass slides in an air and in a vacuum chamber at about 5 × 10^−5^ Torr pressure. The procedure of thermal poling is described as follows: the slides with pressed (glassy carbon) electrodes are heated up to 300 °C and DC voltage of 800 V is applied. Duration of the poling corresponded to ~90% drop of the poling current. This mode of poling was chosen because 300 °C temperature is sufficient to activate ionic mobility [10,23], and the application of 800 V to the glass definitely does not result in phase separation observed in soda-lime glasses under higher voltages [26]. Overall, the passed-through glass charge per electrode area was 0.12 C/cm^2^ and 0.328 C/cm^2^ in the case of vacuum and air poling, respectively. It should be mentioned that air poling with pressed electrodes does not completely isolate anodic surface of a glass slide from atmosphere and should not be considered as close anode poling. In contrast, vacuum poling with pressed electrodes corresponds to close anode configuration, because no species can penetrate into the glass in this case. After the poling, the glasses were subjected to 20 min ion-exchange processing, as described elsewhere [27]: the slides were immersed into Ag_0.05_Na_0.95_NO_3_ (in wt %) melt heated to 325 °C. It has been found that vacuum poling, contrary to air poling, results in a brownish coloration of the glasses after the processing.

To characterize the poled glasses before and after the ion-exchange, we measured their optical absorption spectra in the poled (colored) and unpoled (transparent) regions of the glass using UV-VIS spectrophotometer Specord 50 (Analytik Jena, Jena, Germany). Additionally, we performed a chemical etching of the ion-exchanged glass poled in vacuum in 45% KOH water solution heated to 60 °C and in a room-temperature dilute solution of NH_4_F with the addition of HF: HF (5 µL):NH_4_F (5 g):H_2_O (40 mL). After each iteration of the etching, we measured the optical absorption spectrum of the sample and the height difference between etched and non-etched (protected) areas of the poled glass using stylus profilometer AmBios XP-1 (Ambios Technology Inc., Santa Cruz, CA, USA). Finally, the silver profile of the ion-exchanged glass poled in vacuum was obtained by energy-dispersive X-ray spectroscopy (EDS) of the sample cross-section using Ultim Max 100 (Oxford Instruments, Oxford, UK) system combined with a scanning electron microscope Supra 25 (Zeiss, Jena, Germany).

## 3. Results and Discussion

**Figure 1:**
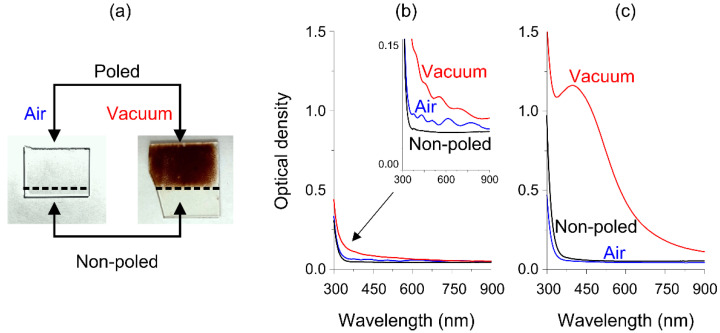
(**a**) Photographs of the ion-exchanged glasses poled prior the ion-exchange. The border between poled and unpoled glass regions is marked with a dash line, and the air- and vacuum-poled samples are indicated “Air” and “Vacuum”, respectively. (**b**) Optical absorption spectrum of non-poled glass and air- and vacuum-poled glasses before and (**c**) after the ion-exchange.

Generally, during the ion-exchange processing of sodium containing silicate glasses, sodium ions in glass are replaced with silver ions from the melt, which results in a small red shift of UV optical absorption edge [28]. The thermal poling in close anode configuration, in turn, results in the formation of a subsurface glass layer depleted with movable ions, first of all sodium ions, and, therefore, inhibits silver ions ion-exchange diffusion into the anodic side of the glass [12,29]. Thus, one can expect that the optical absorption of the vacuum-poled glass remains almost unchanged after the ion-exchange and the glass stays transparent as do air-poled and non-poled ion-exchanged glasses. Surprisingly, after the ion-exchange processing of the vacuum-poled soda-lime glass, we observed a brownish coloration of the glass, contrary to the air-poled one. The photographs of the vacuum-poled and air-poled poled glasses after the ion-exchange are demonstrated in Figure 1a. Measured optical absorption spectra of the poled glasses before and after the ion-exchanges are presented in Figure 1b,c.

Considering the optical absorption spectra of the vacuum- and air-poled glasses before the ion-exchange (Figure 1b), one can see the oscillations in optical density. These oscillations take place because of the formation of a buried Ca-enriched layer in glasses, which always exists in the case of close-anode poling [15] and appears if thermal poling is performed in intermediate, neither close nor completely open, poling configuration [11,29]. The latter takes place in poling with pressed anode in atmosphere in case of insufficient hydronium inflow [11,29]. The oscillations are due to light reflection from the back interface of the Ca-enriched layer [30]. Thus, the burial depth of the back interface of the Ca-enriched layer and the average refractive index of the poled region of the glass can be deduced from the oscillations’ period according to the procedure described elsewhere [30]. We followed the procedure and processed the absorption spectra of the poled glass samples. The estimated burial depths and refractive index (at 525 nm wavelength) of Ca-rich layers for the vacuum-poled and in air-poled glasses were 820 ± 20 nm and 1.596, and 920 ± 50 nm and 1.573, respectively. These values are in coincidence with recent data [31] reported for a similar glass poled with pressed electrodes. One can see that the depth obtained for the vacuum-poled glass is 100 nm less and the refractive index is higher by 0.023 than those for the glass poled in air. This indicates a more efficient accumulation of Ca ions under the anode surface of the glass with vacuum poling because of the absence of inflowing hydronium.

Optical absorption spectrum of the air-poled glass subjected to the ion-exchange demonstrates weak differences relative to the spectrum prior to the ion-exchange, see Figure 1c. This could be due to silver diffusion to the cathode side of the glass, which contains sodium and some weak hydronium-to-sodium exchange at the anode side. The spectrum of the vacuum-poled glass after the ion-exchange is also presented in Figure 1c and shows a high peak of absorption at ~400 nm. Moreover, performed EDS measurement of the poled and ion-exchanged glass sample demonstrates the presence of silver in the depth of the glass. Therefore, we attributed the peak at ~400 nm to the surface plasmon resonance (SPR) absorption of silver nanoparticles (NPs). It is essential that the SPR remained in the absorption spectra after careful cleaning of the samples to exclude formation of NPs on the glass surface [32]. This also gave us the evidence of the formation of silver NPs within the glass. The formation of silver NPs and the corresponding absorption at SPR wavelength at ~400 nm explain the observed brownish color of the samples. The brown rather than yellow, corresponding to the wavelength of 400 nm, color can be explained by the relatively wide absorption peak (see Figure 1c). In addition, an intense brown color indicates essential light absorption by silver NPs, optical density ~0.5 even at 600 nm wavelength.

**Figure 2:**
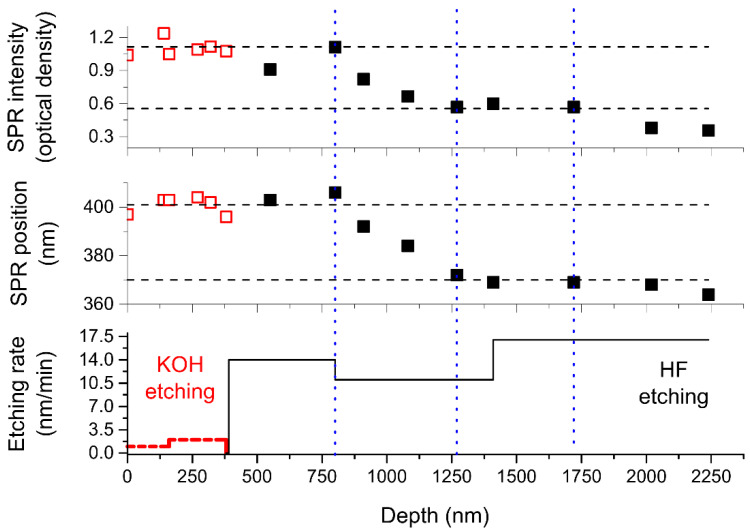
The dependencies of the intensity (**upper**) and spectral position (**middle**) of SPR of silver NPs formed in vacuum-poled glass after the ion-exchange, and etching rate (**lower**) of the glass on distance from the sample surface. Hollow squares/dashed lines correspond to KOH etching, and black squares / solid lines to NH_4_F/HF etching. The etching rate of ~17 nm/min (starts from ~1400 nm) corresponds to the non-poled glass after the ion-exchange.

To determine the localization of formed silver NPs we successively etched glass poled in a vacuum in KOH and NH_4_F/HF water solutions (see Section 2). Note, KOH solution preferably etches silica, while NH_4_F/HF solution etches multicomponent silicate glasses, and the etching rate depends on the type and content of modifiers in the glass (for soda-lime glass, these are alkali and alkaline-earth metals). The measured etching rate of virgin soda-lime glass used in the experiments was about zero in KOH solution and ~17 nm/min in NH_4_F/HF solution. In Figure 2, we present the dependencies of the intensity and spectral position of SPR, and the etching rate on distance from the sample surface.

One can see in Figure 2, the NPs SPR intensity and spectral position do not change until 800 nm of the glass is etched off. This depth exactly coincides with the location of the back interface of the Ca-enriched layer calculated via the oscillations in the optical absorption spectrum presented in Figure 1. The drop in the etching rate from 14 nm/min to 11 nm/min at ~800 nm depth also evidences compositional changes in the glass. As was reported in [15,33], in the glasses poled with the absence of hydrogenated species in-flow, the Ca-enriched layer is followed by a layer enriched with hydrogen. Hydrogen comes from the residual hydroxyl, which presents in partially leached in the atmosphere glass surface [34].

Removal of the next 470 nm (the 1250 nm glass layer is etched off in total) leads to a decrease in the SPR intensity by half of the initial value and in the blue shift of SPR position from 400 to 370 nm. Taking into account the nanoparticles’ shape, we attributed the peaks at ~370 and 400 nm to the SPR of silver NPs with a diameter of ~3–6 nm [35] (“smaller” NPs) and ~5–10 nm [36,37] (“bigger” NPs), respectively. Following etching of 450 nm glass layer (1700 nm is etched off in total) does not influence either SPR intensity, or its spectral position. At this point, all poling-modified glass region is etched off, and the etching rate of 17 nm/min corresponds to the one we measured for a virgin glass. Subsequent etching results in vanishing of SPR from the optical absorption spectra.

All of the above allowed us to conclude that ~750 nm is the depth starting from which “bigger” silver nanoparticles present in the glass and their concentration drops in the region from 750 to 1250 nm from the surface, while “smaller” NPs still present. It is worth noting that this NPs containing region is only slightly thicker and deeper from the surface than the region from ~600 to ~1000 nm where essential gradients of concentration of movable ions were registered with secondary ions mass spectrometry in the same glass poled at the same temperature, but under a slightly less voltage (700 V) [38]. In addition, this region begins very close to the determined depth corresponding to the back interface of the Ca-enriched glass region (~820 nm), where calcium concentration begins to change.

It should be mentioned here that in accordance with the existing modeling and experimental data [39,40], spatial charge formed in poling should be concentrated in the areas of concentration gradients. This charge is negative [15] and should be attributed to the negatively charged non-bridging oxygen atoms which lost bonded cations because of the electric field effect [39]. The latter allows an interpretation of the observed reduction and clustering of silver ions penetrated into the vacuum-poled glass during the ion-exchange. Supposedly, these oxygen atoms donate electrons to silver ions diffusing and drifting under the field of the frozen charge towards the spatial charge region, and thermal diffusion of reduced (neutral) silver atoms leads to their clustering and the growth of silver nanoparticles. The concentration of neutral silver is higher in the region of spatial charge where silver reduction occurs and silver atoms accumulate, and the concentration decreases when silver atoms diffuse deeper in the glass. This is the reason of the formation of “bigger” NPs in the area of concentration gradients and “smaller “NPs in the region deeper, starting from ~1250 nm to ~1750 nm.

**Figure 3:**
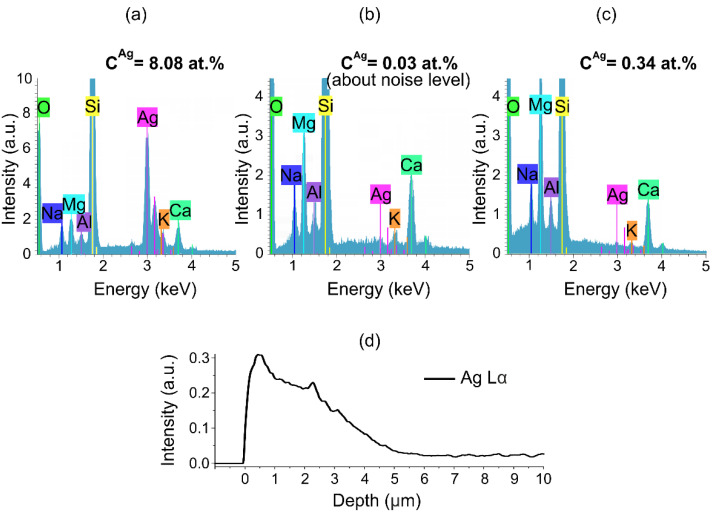
EDS spectra of (**a**) non-poled ion-exchanged glass surface, (**b**) vacuum-poled and ion-exchanged glass surface before and (**c**) after ~400 nm layer was etched off. (**d**) The dependence of normalized intensity of Ag Lα line (normalization by intensity of Si Kα line) on distance from the surface of poled sample.

The accumulation of silver atoms in the spatial charge region should also provide a higher silver EDS signal from this region and area below where silver NPs have grown. This was confirmed by EDS measurements using normal incidence of e-beam to the sample surface, which show that the concentration of silver on the surface of the sample obtained by vacuum poling and ion exchange is essentially less than the concentration of silver on the surface of this sample after KOH etching, resulting in the removal of ~400 nm layer (see Figure 3b,c). It is worth noting that after ion exchange in the non-poled glass, the concentration of silver is almost two orders of magnitude higher (see Figure 3a).

Unfortunately, the resolution of the scanning electron microscope did not allow observing nanoparticles less than 10 nm in size, and in interpreting the experimental results, we rely on the SPR typical for silver NPs. Nevertheless, the comparison of EDS-measured at the sample cut silver concentration profile (see Figure 3d) shows that the depth of silver penetration in the sample is about 5 µm, which exceeds the depth at which SPR amplitude and, respectively, NPs concentration essentially drop. Thus, we can conclude that silver in ionic or neutral state and, possibly, partly in the form of silver molecular or ionic clusters, is present in the sample after the ion-exchange up to the depth of about 5 µm. In contrast, NPs are formed only in the spatial charge region, up to 1700 nm. Thus, it can be concluded that both spatial charge and silver ions are responsible for the formation of NPs.

## 4. Conclusions

Finally, it is demonstrated for the first time that the vacuum poling of soda-lime silicate glass and following processing of the glass in the melt containing silver ions results in the formation of silver nanoparticles in the subanodic region of the glass. The nanoparticles are about 3–10 nm in size and are buried beneath the surface of the glass at the depth of 800–1700 nm. This phenomenon is unique for vacuum-poled glasses.

It is noteworthy that the region of the nanoparticles formation inside the glass corresponds to the location of the uncompensated spatial charge formed by poling. This makes it possible to associate the nanoparticles formation with the transfer of electrons from negatively charged non-bridging oxygen atoms to silver ions. The nanoparticles do not form in the area just beneath the glass surface, which is depleted in ions of metals by the poling, despite the fact that the removal of positive metal ions should lead to the formation of an uncompensated negative space charge (non-bridging oxygen). This indicates the absence of the spatial charge in that region of the vacuum-poled glass. In consequence, this allows stating that the neutralization of glass by switching non-bridging oxygen bonds to bridging ones, which is confirmed by the well-known phenomenon of oxygen release in poled glasses, should occur in parallel with the movement of calcium, magnesium, and sodium ions into the depth of the glass.

## Figures and Tables

**Table 1:** Composition of Menzel glass in wt % of oxides.

SiO_2_	Na_2_O	CaO	MgO	K_2_O	Al_2_O	Others
72.2	14.3	6.4	4.3	1.2	1.2	0.33

## Data Availability

The data presented in this study are available on request from the corresponding author.

## References

[1] Lind F., Palles D., Möncke D., Kamitsos E.I., Wondraczek L. (2017). Modifying the surface wetting behavior of soda-lime silicate glass substrates through thermal poling. J. Non-Cryst. Solids.

[2] Lepicard A., Cardinal T., Fargin E., Adamietz F., Rodriguez V., Richardson K., Dussauze M. (2015). Surface Reactivity Control of a Borosilicate Glass Using Thermal Poling. J. Phys. Chem. C.

[3] Lepicard A., Cardinal T., Fargin E., Adamietz F., Rodriguez V., Richardson K., Dussauze M. (2016). Micro-structuring the surface reactivity of a borosilicate glass via thermal poling. Chem. Phys. Lett..

[4] Kamenskii A.N., Reduto I.V., Petrikov V.D., Lipovskii A.A. (2016). Effective diffraction gratings via acidic etching of thermally poled glass. Opt. Mater..

[5] Ikutame N., Kawaguchi K., Ikeda H., Sakai D., Harada K., Funatsu S., Nishii J. (2013). Low-temperature fabrication of fine structures on glass using electrical nanoimprint and chemical etching. J. Appl. Phys..

[6] Ziemath E.C., Araújo V.D., Escanhoela C.A. (2008). Compositional and structural changes at the anodic surface of thermally poled soda-lime float glass. J. Appl. Phys..

[7] Dussauze M., Kamitsos E.I., Fargin E., Rodriguez V. (2009). Refractive index distribution in the non-linear optical layer of thermally poled oxide glasses. Chem. Phys. Lett..

[8] Luo J., Bae S., Yuan M., Schneider E., Lanagan M.T., Pantano C.G., Kim S.H. (2018). Chemical structure and mechanical properties of soda lime silica glass surfaces treated by thermal poling in inert and reactive ambient gases. J. Am. Ceram. Soc..

[9] Paraillous M., Dussauze M., Fargin E., Poulon-Quintin A., Cardinal T. (2016). Hardness reinforcement by surface engineering of soda lime silicate glass under thermal poling. Proceedings of the Photonics and Fiber Technology 2016 (ACOFT, BGPP, NP).

[10] Redkov A.V., Melehin V.G., Statcenko V.V., Lipovskii A.A. (2015). Nanoprofiling of alkali-silicate glasses by thermal poling. J. Non-Cryst. Solids.

[11] An H., Fleming S. (2006). Second-order optical nonlinearity and accompanying near-surface structural modifications in thermally poled soda-lime silicate glasses. J. Opt. Soc. Am. B.

[12] Dergachev A., Kaasik V., Lipovskii A., Melehin V., Redkov A., Reshetov I., Tagantsev D. (2020). Control of soda-lime glass surface crystallization with thermal poling. J. Non-Cryst. Solids.

[13] Karam L., Adamietz F., Michau D., Gonçalves C., Kang M., Sharma R., Murugan G.S., Cardinal T., Fargin E., Rodriguez V. (2020). Electrically Micro-Polarized Amorphous Sodo-Niobate Film Competing with Crystalline Lithium Niobate Second-Order Optical Response. Adv. Opt. Mater..

[14] Dussauze M., Rodriguez V., Lipovskii A., Petrov M., Smith C., Richardson K., Cardinal T., Fargin E., Kamitsos E.I. (2010). How does thermal poling affect the structure of soda-lime glass?. J. Phys. Chem. C.

[15] Lepienski C.M., Giacometti J.A., Leal Ferreira G.F., Freire F.L., Achete C.A. (1993). Electric field distribution and near-surface modifications in soda-lime glass submitted to a dc potential. J. Non-Cryst. Solids.

[16] Dussauze M., Fargin E., Lahaye M., Rodriguez V., Adamietz F. (2005). Large second-harmonic generation of thermally poled sodium borophosphate glasses. Opt. Express.

[17] Moura A.L., de Araujo M.T., Vermelho M.V.D., Aitchison J.S. (2006). Improved stability of the induced second-order nonlinearity in soft glass by thermal poling. J. Appl. Phys..

[18] Chen H.Y., Lin H.Y. (2019). Thermal poling induced second-order optical nonlinearity in phosphosilicate glass thin films. J. Mod. Opt..

[19] Yudistira D., Faccio D., Corbari C., Kazansky P.G., Benchabane S., Pruneri V. (2008). Electric surface potential and frozen-in field direct measurements in thermally poled silica. Appl. Phys. Lett..

[20] Alvarado R., Karam L., Dahmani R., Lepicard A., Calzavara F., Piarristeguy A., Pradel A., Cardinal T., Adamietz F., Fargin E. (2020). Patterning of the Surface Electrical Potential on Chalcogenide Glasses by a Thermoelectrical Imprinting Process. J. Phys. Chem. C.

[21] Scherbak S.A., Kaasik V.P., Zhurikhina V.V., Lipovskii A.A. (2021). SEM-visualization of a spatial charge and a giant potassium peak in a corona-poled glass. J. Phys. Condens. Matter.

[22] Doremus R.H. (2005). Mechanism of electrical polarization of silica glass. Appl. Phys. Lett..

[23] Redkov A.V., Melehin V.G., Lipovskii A.A. (2015). How Does Thermal Poling Produce Interstitial Molecular Oxygen in Silicate Glasses?. J. Phys. Chem. C.

[24] Von Hippel A., Gross E.P., Jelatis J.G., Geller M. (1953). Photocurrent, Space-Charge Buildup, and Field Emission in Alkali Halide Crystals. Phys. Rev..

[25] Microscope Slides. www.agarscientific.com/microscope-slides.html.

[26] An H., Fleming S.C. (2006). Near-anode phase separation in thermally poled soda lime glass. Appl. Phys. Lett..

[27] Liñares J., Sotelo D., Lipovskii A.A., Zhurihina V.V., Tagantsev D.K., Turunen J. (2000). New glasses for graded-index optics: Influence of non-linear diffusion in the formation of optical microstructures. Opt. Mater..

[28] Spierings G.A.C.M. (1987). Optical absorption of Ag^+^ ions in 11(Na,Ag)_2_O · 11B_2_O_3_ · 78SiO_2_ glass. J. Non-Cryst. Solids.

[29] Babich E., Reduto I., Redkov A., Reshetov I., Zhurikhina V., Lipovskii A. (2020). Thermal poling of glasses to fabricate masks for ion exchange. J. Phys. Conf. Ser..

[30] Babich E., Raskhodchikov D., Lubyankina E., Lipovskii A. (2021). Depth of glass poling—Via optical transmission spectra. Optik.

[31] Fabijanić I., Pervan P., Okorn B., Sancho-Parramon J., Janicki V. (2020). Ellipsometry-based study of glass refractive index depth profiles obtained by applying different poling conditions. Appl. Opt..

[32] Zhurikhina V.V., Brunkov P.N., Melehin V.G., Kaplas T., Svirko Y., Rutckaia V.V., Lipovskii A.A. (2012). Self-assembled silver nanoislands formed on glass surface via out-diffusion for multiple usages in SERS applications. Nanoscale Res. Lett..

[33] Smith N.J., Pantano C.G. (2014). Structural and compositional modification of a barium boroaluminosilicate glass surface by thermal poling. Appl. Phys. A Mater. Sci. Process..

[34] Majérus O., Lehuédé P., Biron I., Alloteau F., Narayanasamy S., Caurant D. (2020). Glass alteration in atmospheric conditions: Crossing perspectives from cultural heritage, glass industry, and nuclear waste management. NPJ Mater. Degrad..

[35] Balavandy S.K., Shameli K., Biak D.R.B.A., Abidin Z.Z. (2014). Stirring time effect of silver nanoparticles prepared in glutathione mediated by green method. Chem. Cent. J..

[36] Chen Y., Karvonen L., Säynätjoki A., Ye C., Tervonen A., Honkanen S. (2011). Ag nanoparticles embedded in glass by two-step ion exchange and their SERS application. Opt. Mater. Express.

[37] Sancho-Parramon J., Janicki V., Dubček P., Karlušić M., Gracin D., Jakšić M., Bernstorff S., Meljanac D., Juraić K. (2010). Optical and structural properties of silver nanoparticles in glass matrix formed by thermal annealing of field assisted film dissolution. Opt. Mater..

[38] Kaasik V.P., Lipovskii A.A., Raskhodchikov D.V., Reshetov I.V., Tagantsev D.K. (2019). How to reveal the correct elemental concentration profiles in poled multicomponent silicate glasses from the data of secondary ion mass spectrometry (SIMS). J. Non-Cryst. Solids.

[39] Petrov M.I., Lepen’kin Y.A., Lipovskii A.A. (2012). Polarization of glass containing fast and slow ions. J. Appl. Phys..

[40] Xu W., Arentoft J., Wong D., Fleming S. (1999). Evidence of space-charge effects in thermal poling. IEEE Photonics Technol. Lett..

